# The proline-rich domain of tau plays a role in interactions with actin

**DOI:** 10.1186/1471-2121-10-81

**Published:** 2009-11-08

**Authors:** Hai Jin He, Xing Sheng Wang, Rong Pan, Dong Liang Wang, Ming Nan Liu, Rong Qiao He

**Affiliations:** 1 State Key Laboratory of Brain and Cognitive Sciences, Institute of Biophysics, Key Lab of Mental Health, Institute of Psychology, Chinese Academy of Sciences, Beijing, PR China; 2 Graduate University, Chinese Academy of Sciences, Beijing, PR China

## Abstract

**Background:**

The microtubule-associated protein tau is able to interact with actin and serves as a cross-linker between the microtubule and actin networks. The microtubule-binding domain of tau is known to be involved in its interaction with actin. Here, we address the question of whether the other domains of tau also interact with actin.

**Results:**

Several tau truncation and deletion mutants were constructed, namely N-terminal region (tauN), proline-rich domain (tauPRD), microtubule binding domain (tauMTBD) and C-terminal region (tauC) truncation mutants, and microtubule binding domain (tauΔMTBD) and proline-rich domain/microtubule binding domain (tauΔPRD&MTBD) deletion mutants. The proline-rich domain truncation mutant (tauPRD) and the microtubule binding domain deletion mutant (tauΔMTBD) promoted the formation of actin filaments. However, actin assembly was not observed in the presence of the N-terminal and C-terminal truncation mutants. These results indicate that the proline-rich domain is involved in the association of tau with G-actin. Furthermore, results from co-sedimentation, solid phase assays and electron microscopy showed that the proline-rich domain is also capable of binding to F-actin and inducing F-actin bundles. Using solid phase assays to analyze apparent dissociation constants for the binding of tau and its mutants to F-actin resulted in a sequence of affinity for F-actin: tau >> microtubule binding domain > proline-rich domain. Moreover, we observed that the proline-rich domain was able to associate with and bundle F-actin at physiological ionic strength.

**Conclusion:**

The proline-rich domain is a functional structure playing a role in the association of tau with actin. This suggests that the proline-rich domain and the microtubule-binding domain of tau are both involved in binding to and bundling F-actin.

## Background

Tau is an important microtubule-associated protein, promoting microtubule assembly and stabilizing microtubules [1-3]. The protein is recognized as a multifunctional molecule that interacts with actin in addition to microtubules [4-12], and is involved in the organization of the cytoskeletal network [4,5]. Actin monomers (G-actin) were found to form gels in the presence of tau [8]. According to Farias and colleagues [5], the association of tau with tubulin immobilized on a solid phase support system is inhibited by actin monomers, and a higher inhibition can be attained with preassembled actin filaments. Interestingly, tau can interact with F-actin, resulting in bundles of F-actin. MacLean-Fletcher and Pollard [6] have observed that tau dramatically induces an increase in the viscosity of actin filaments. Using electron microscopy tau has been shown to be capable of bundling microfilaments. Examination of morphological aspects of microtubules and actin filaments which coexist *in vitro *revealed associations between both cytoskeletal filaments, and in some cases, the presence of fine filamentous structures bridging these polymers [7]. Several reports have demonstrated that tau interacts with actin *in vivo*. Sub-portions of tau co-immunoprecipitated with actin filaments have been found in various cell types [4]. As described by Yu and colleagues [13], under NGF stimulation, tau is distributed at the ends of cellular extensions, where it associates with actin in a microtubule-independent manner in PC12 cells. Moreover, Fluga and co-workers [14] have provided evidence that tau induces changes in the organization and stability of neuronal actin filaments, which in turn contributes to Alzheimer's-like neurodegeneration in *Drosophila *and mouse model systems. This further demonstrates the physiological importance of interactions between tau and actin.

According to Buee and colleagues [15], tau consists of four parts: the N-terminal region, the proline-rich domain (PRD), the microtubule-binding domain (MTBD) and the C-terminal region. The microtubule binding domain has been reported to bind to actin [7,9], but no data is available for the other regions bound to actin. It has been proposed that the proline-rich domain of tau participates in interactions with microtubules [16-18]. Interactions between tau and DNA have been studied in our laboratory [19], and PRD and MTBD were found to associate cooperatively with the minor groove in DNA double strands. These results intrigued and led us to investigate whether the proline-rich domain of tau also participates in interactions with actin.

## Results

### Tau binds to G-actin and F-actin from skeletal muscle and platelets

Human actin has three subtypes, alpha actin being found primarily in muscle, and beta and gamma actin in other tissues. The interaction of tau with alpha actin has been well studied, however, little attention has been given to beta and gamma actin. Since tau mainly exists in neurons, beta and gamma actin are the subtypes of actin that tau can encounter. In this work we mainly used skeletal muscle actin. Platelet actin (a mixture of beta and gamma actin) was also employed to test whether subtypes of actin differ in their interactions with tau.

**Figure 1:**
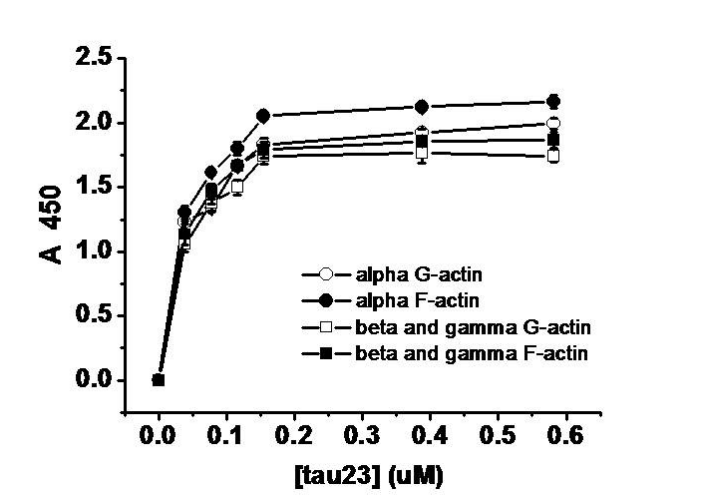
**Binding of tau to actin detected by solid phase assay**. Wells were coated with 50 μl G-actin and F-actin (5 μg/ml) from skeletal muscle and platelets, respectively. Aliquots of different concentrations of tau (from 0.03 to 0.55 μM) were incubated with actin in binding buffer (50 mM Hepes containing 0.5 mM EGTA and 0.5 mM MgCl_2_, pH 7.5). Immunoreactivity was monitored in the solid phase assay using secondary antibody-labelled HRP. Each point represents the mean of five determinations.

Here, solid phase assays were used to study interactions between tau and actin. Human tau23 (352 aa) was employed in our experiments. G-actin and F-actin from skeletal muscle and platelets were immobilized in 96-well plates, and increasing concentrations of tau were added. Levels of bound tau were monitored by using an anti-tau monoclonal antibody. As illustrated in Figure 1, absorbance increased with increasing tau concentration, indicating a positive correlation between added tau and bound tau. BSA used as a negative control showed no interaction with G-actin or F-actin (data not shown). These results suggest that tau is capable of binding to G-actin and F-actin.

As shown in Table 1, the data could be fitted to a one-site binding model. Kapp and Bmax values for the binding of G-actin and F-actin to tau showed close agreement, suggesting that polymerization of G-actin does not affect its binding to tau. Similar patterns were observed for the binding of tau to G-actin and F-actin from both skeletal muscle and platelets.

**Table 1:** Biochemical Binding Parameters from Solid Phase Assays

actin	Bmax (μM)	Kapp (μM)	R
α-G-	2.10 ± 0.07	0.031 ± 0.007	0.99
α-F-	2.31 ± 0.05	0.030 ± 0.004	0.99
β/γ-G-	1.90 ± 0.06	0.029 ± 0.005	0.99
β/γ-F-	2.01 ± 0.04	0.027 ± 0.003	0.99

Conditions were the same as those referred to in Figure 1. Binding parameters were estimated with the non-linear curve fit Hyperbl function (ΔA_450 _= Bmax * [tau]/(Kapp+ [tau]) in the Origin program.

### The proline-rich domain is involved in binding to G-actin

**Figure 2:**
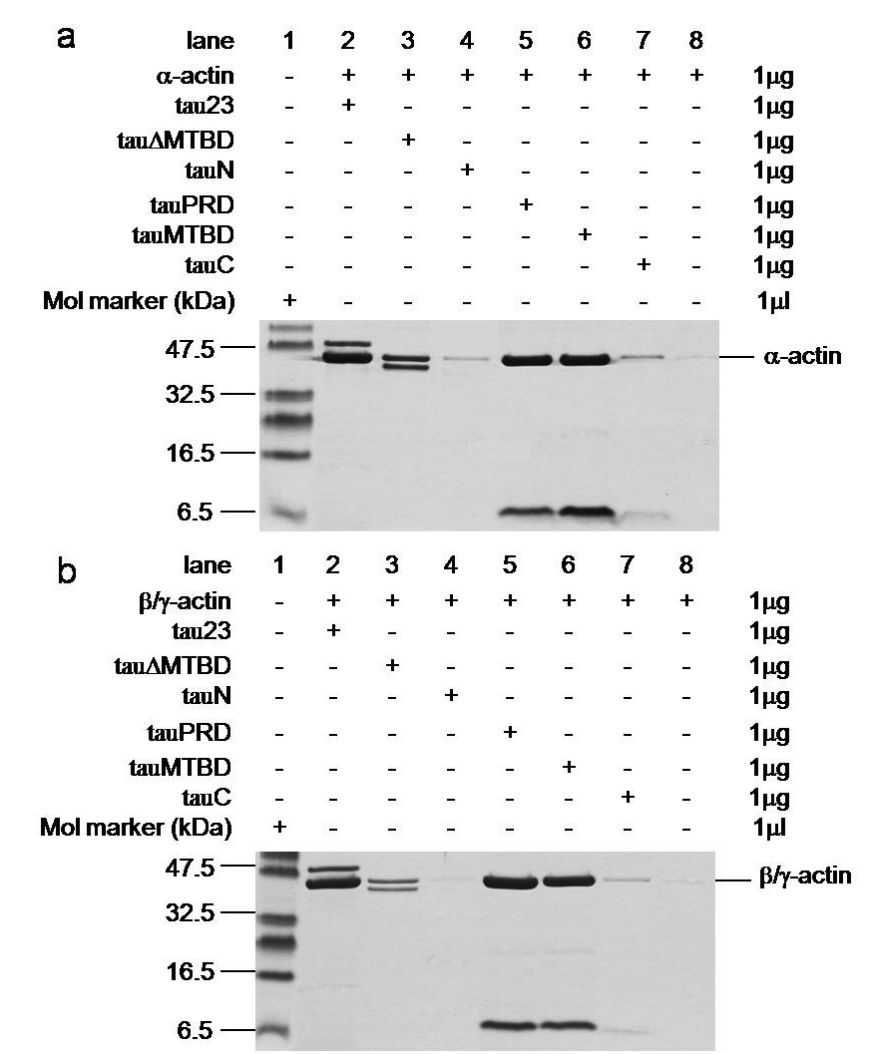
**Low speed co-sedimentation assay of G-actin in the presence of tau and tau mutants**. Tau and tau mutants were incubated with G-actin in a 1:1 mass ratio at 37°C in binding buffer and then centrifuged (25,000 g, 4°C, 30 min). Before electrophoresis on gels, pellets were resuspended with 20 mM Tris-HCl (pH 8.0) and boiled for 5 min. Rabbit skeletal muscle G-actin (panel a) and human platelet G-actin (panel b) were used. Actin alone was used as negative control. Different concentrations of tauΔPRD&MTBD reacted with G-actin. The pellets were electrophoresed on 12% SDS-PAGE gels after the centrifugation (panel c).

**Figure 3:**
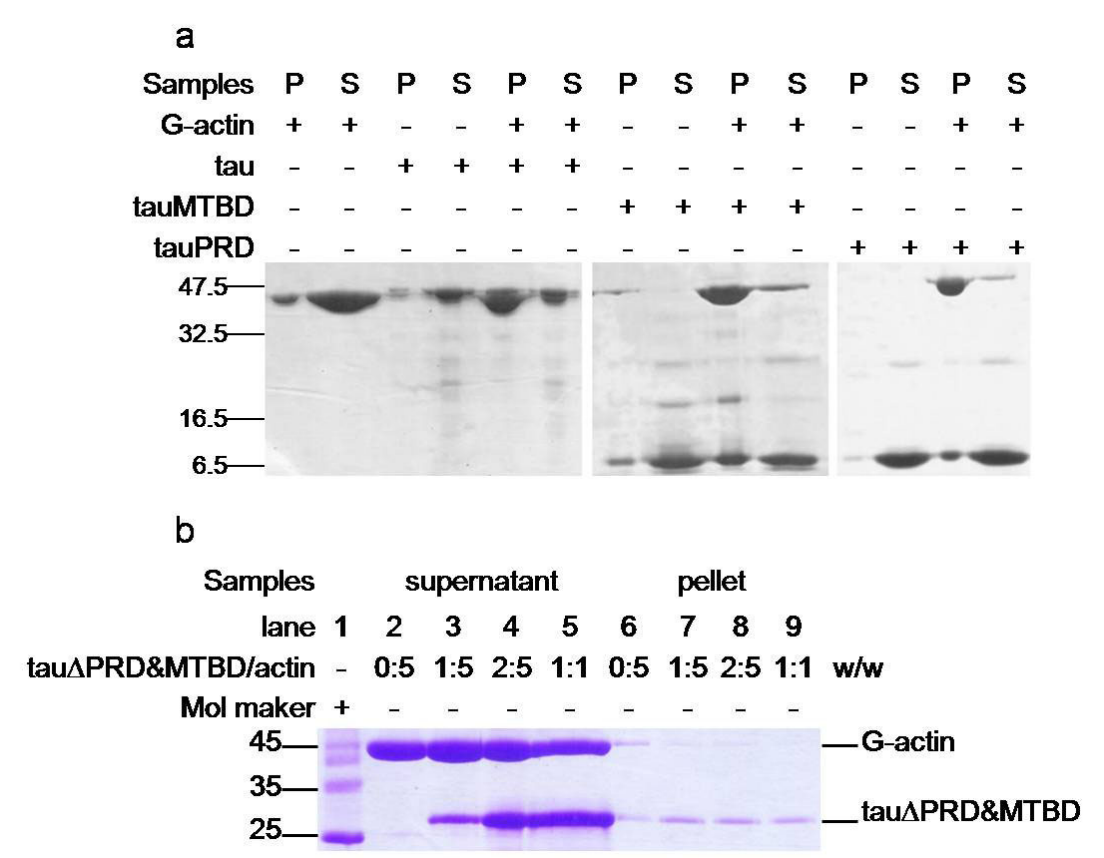
**G-actin in the presence of tau mutants at low speed co-sedimentation assay**. Conditions were referred to Figure 2, G-actin (4 μg) was incubated with equal amount of tauRPD, tauMTBD, tau (panel a), and with tauΔPRD&MTBD (panel b) in binding buffer for 20 min in 37°C, followed by low-speed centrifugation. The pellet fraction (P) and supernatant fraction (S) were loaded in gels.

**Figure 4:**
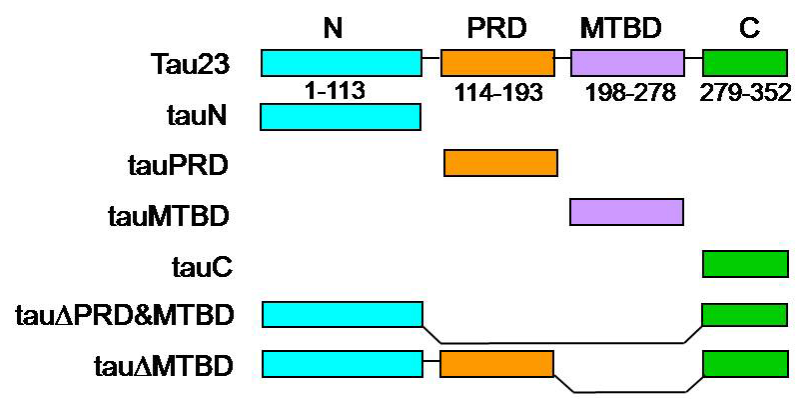
**Construction of tau and its mutants**. Six truncated tau isolation and deletion mutants were constructed: tauN (N-terminal region, 1-113), tauPRD (proline-rich domain, 114-193, containing 22 proline residues), tauMTBD (microtubule-binding domain, 198-278) and tauC (C-terminal region, 279-352). TauΔMTBD is a mutant in which the MTBD domain is deleted. TauΔPRD&MTBD is a mutant in which both PRD and MTBD are deleted.

Co-sedimentation assays are commonly used to investigate the binding of specific proteins or protein domains with actin [8]. Low speed centrifugation (25,000 g) can only pellet F-actin bundles, while high speed centrifugation (100,000 g) can pellet all F-actin filaments. Under our experimental conditions, tau could co-sediment with actin by low speed centrifugation (Figure 2; panel a, Figure 3). This result indicated that tau can both induce polymerization of G-actin to F-actin and to F-actin bundles. To investigate the roles of the four regions of tau in interactions with actin, we designed a group of primers (Additional file 1) and constructed a series of tau truncation and deletion mutants: mutants in which the N-terminal region (tauN), proline-rich domain (tauPRD), microtubule-binding domain (tauMTBD), C-terminal region (tauC) were separated, and mutants in which the MTBD (tauΔMTBD), and both the MTBD and PRD domains (tauΔPRD&MTBD) were deleted (Figure 4).

Results indicated that the deletion of MTBD did not eliminate the ability of tau to bind to actin since both ΔMTBD and G-actin were present in protein pellets as shown by SDS-PAGE (panel a, Figure 2). Under our experimental conditions, the tauN, tauC and tauΔPRD&MTBD (panel b, Figure 3) mutants exhibited no association with actin in co-sedimentation assays, indicating that the proline-rich domain may be an essential domain involved in the association of tau with actin. Incubation of the tauPRD mutant with G-actin gave rise to a positive result in co-sedimentation assays, and tauPRD, tauMTBD, tau and BSA alone used as negative controls did not show any deposits in co-sedimentation assays (Additional file 2). To judge the efficiency of bundling during the reaction, both the pellet and supernatant fraction were loaded in gels (panel a, Figure 3). The result showed that most of the G-actin was in the pellet fraction under the experimental conditions, presenting the formation of F-actin bundles. Taken together, these results suggest that the proline-rich domain is involved in the association of tau with G-actin.

Platelet actin was also used in low speed co-sedimentation assays. Results were similar to those obtained for skeletal muscle actin (panel b, Figure 2). Under the same conditions, tauPRD bound to skeletal muscle and platelet actin without discrimination, i.e. tauPRD was capable of binding to alpha-, beta- and gamma-actin. These results again indicate that in addition to the microtubule-binding domain, the proline-rich domain played an important role in the association of tau with actin.

**Figure 5:**
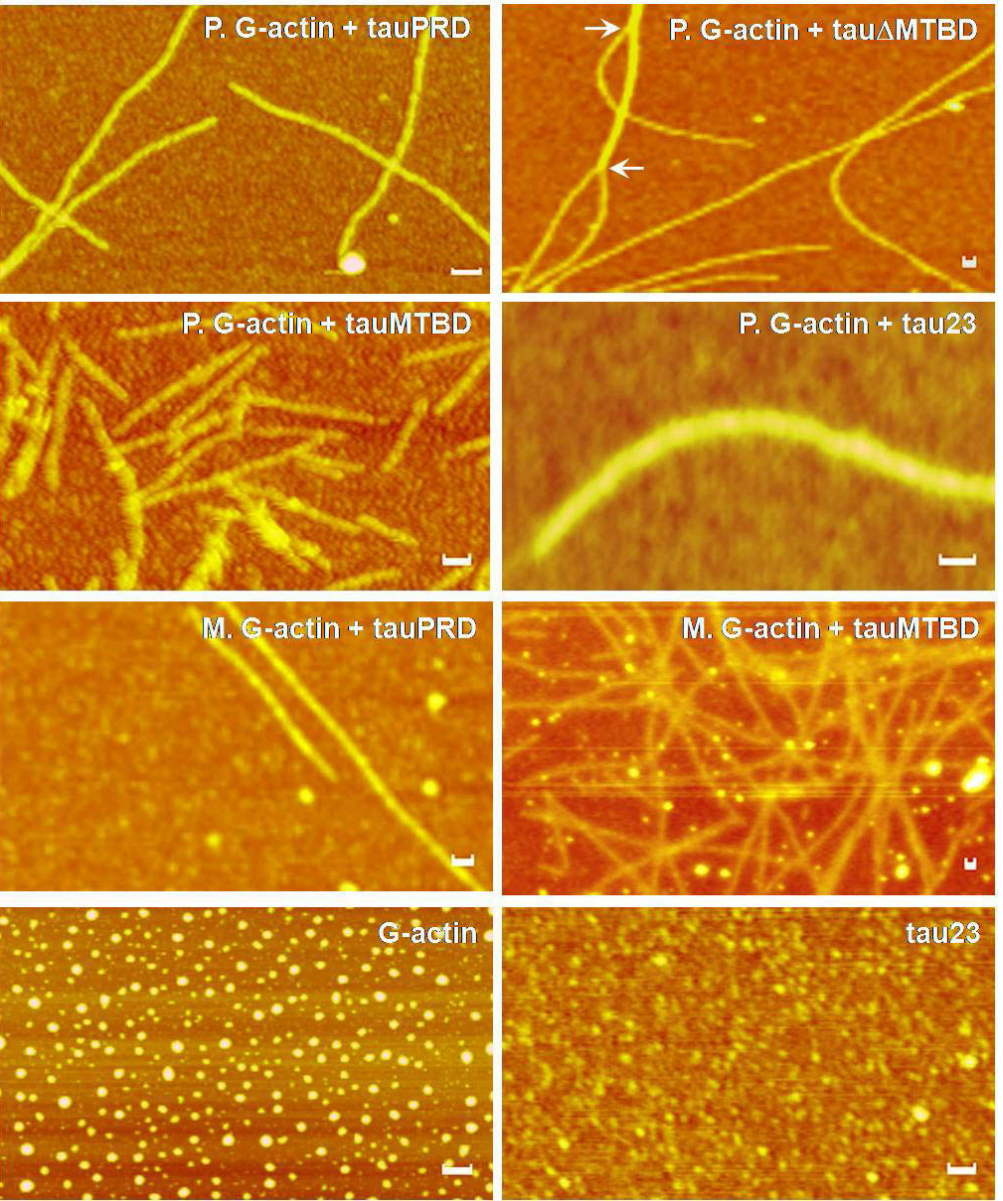
**Interactions of tauPRD with G-actin observed by atomic force microscopy**. Skeletal muscle (M) and platelet (P) G-actin were incubated with tauPRD in binding buffer at 37°C for 30 min and then aliquots were taken for observation by atomic force microscopy as indicated. Bars in panels are 50 nm.

Atomic force microscopy was used to observe the morphology of actin-tauPRD complexes (Figure 5). Filaments were observed after globular platelet actin was incubated with tauPRD. Under the experimental conditions used, tau, tauΔMTBD and tauMTBD also induced formation of F-actin. When tauN, tauC and BSA were incubated separately with G-actin, no filaments were observed (data not shown). G-actin and tau were observed as globular particles in the absence of actin. This demonstrates again that tauPRD is a functional domain involved in the association of tau with actin and in the promotion of G-actin assembly.

### The proline-rich domain binds to F-actin and promotes F-actin bundling

**Figure 6:**
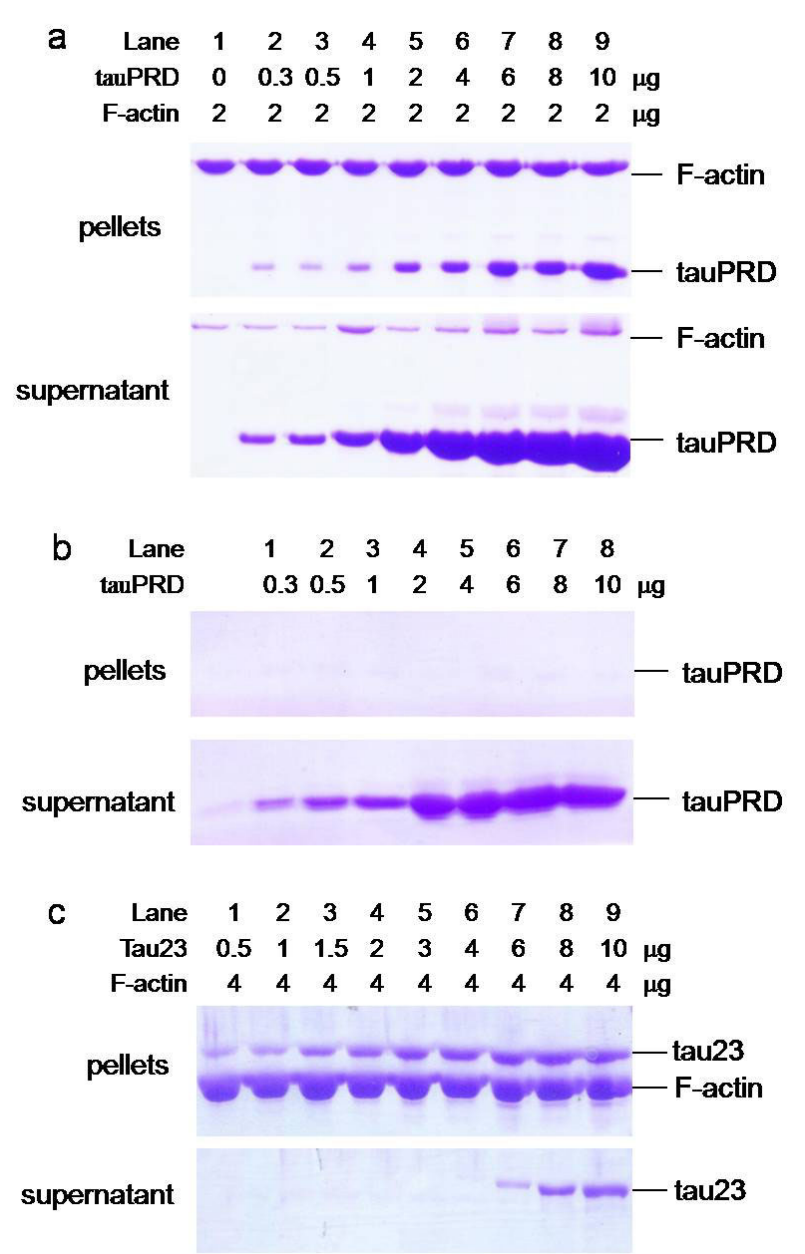
**High speed co-sedimentation assay of tauPRD and F-actin**. F-actin (at a constant concentration) was incubated with different concentrations of tauPRD (panel a), tauPRD alone (panel b) and tau (panel c) in the binding buffer without addition of extra NaCl at 37°C for 40 min and then centrifuged (100,000 g, 4°C, 60 min). The pellet and supernatant were electrophoresed on 12% SDS-PAGE gels.

That tauPRD co-sedimented with actin under low-speed centrifugation suggested the formation of F-actin bundles. To investigate the induction of F-actin bundles, alpha F-actin was incubated with different concentrations of tauPRD, followed by high speed centrifugation to pellet the F-actin-bound tauPRD (panel a, Figure 6). TauPRD alone and tau with F-actin were used as a negative and positive control, respectively (panel b and c, Figure 6). As shown in Figure 6, tauPRD co-sedimented with F-actin, showing that tauPRD binds to F-actin, although its affinity with F-actin was weaker than that of tau.

**Figure 7:**
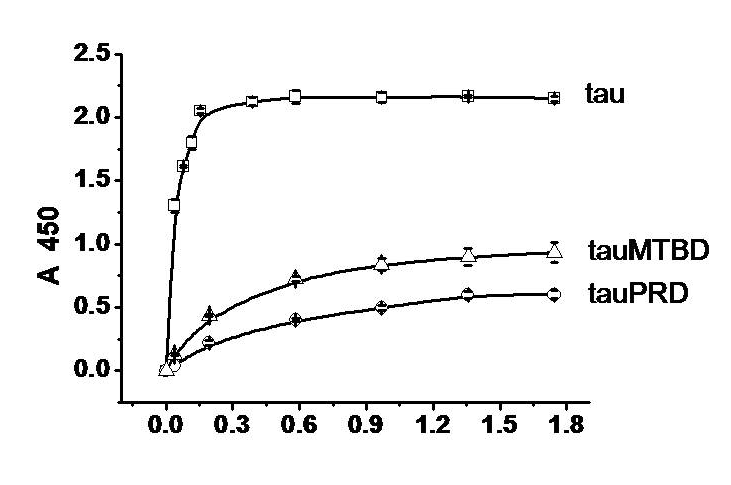
**Solid phase assay studies on the interaction of tauPRD with F-actin**. 50 μl skeletal muscle F-actin (5 μg/ml) was added to solid phase assay wells. Aliquots of different concentrations of tauPRD (○), tau (□) and tauMTBD (△) were incubated with actin in binding buffer. Immunoreactivity was monitored using secondary antibody-labelled HRP. Each point represents the mean of five determinations. Binding parameters were estimated by fitting data using the Hyperbl function in Origin (Table 2).

To confirm the results from co-sedimentation assays, the association of tauPRD, tauMTBD or tau with F-actin was also analyzed by solid phase assay. As shown in Figure 7, tauPRD, tauMTBD and tau could bind to F-actin. Kapp and Bmax values were analyzed as previously (Table 2). The value of Kapp for tauPRD was greater than that for tauMTBD and at least 10 times greater than that for tau (Kapp_tauPRD_: Kapp_tauMTBD_: Kapp_tau _≈ 19:10:1) (Table 2). The value of Bmax_tauPRD _was close to that of Bmax_tauMTBD_, but less than that of Bmax_tau_. Bmax_tau _was approximately equal to the sum of Bmax_tauPRD _and Bmax_tauMTBD_, suggesting that both the PRD and MTBD domains of tau bind to F-actin simultaneously.

**Table 2:** Biochemical Binding Parameters from Solid Phase Assays

Tau	Bmax (μM)	Kapp (μM)	R
tau	2.31 ± 0.05	0.03 ± 0.004	0.99
tauPRD	0.81 ± 0.05	0.57 ± 0.09	0.99
tauMTBD	1.09 ± 0.01	0.29 ± 0.01	0.99

Data were taken from Figure 6, and conditions were the same as for Table 1, except that the reaction of F-actin with tauPRD was monitored by solid phase assay.

**Figure 8:**
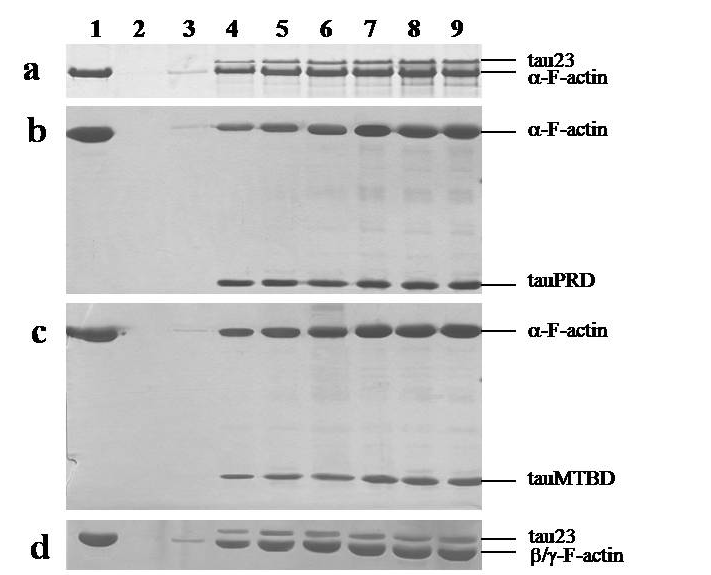
**Low speed co-sedimentation assay of F-actin in the presence of tauPRD**. Conditions were the same as those described for Figure 4, except that F-actin (5 μg) from skeletal muscle (used in panels a, b and c) and platelet actin (used in panel d) were incubated with tau at concentrations of 0, 1, 3, 5, 7, 10, and 15 μM (lanes 3 to 9 in each panel) and with tauPRD or tauMTBD at concentrations of 0, 5, 10, 15, 25, 35, and 45 μM (lanes 3 to 9). After sedimentation at 25,000 g, pellets of bundles were electrophoresed on SDS-PAGE gels. Total amount of F-actin (lane 1) and BSA alone (lane 2) were used as controls.

Low-speed co-sedimentation was used to measure F-actin bundles in the presence of tauPRD. As shown in Figure 8, tauPRD co-sedimented with F-actin, clearly showing that bundles were formed during the reaction. Similar results were observed when tau and tauMTBD were used as positive controls.

**Figure 9:**
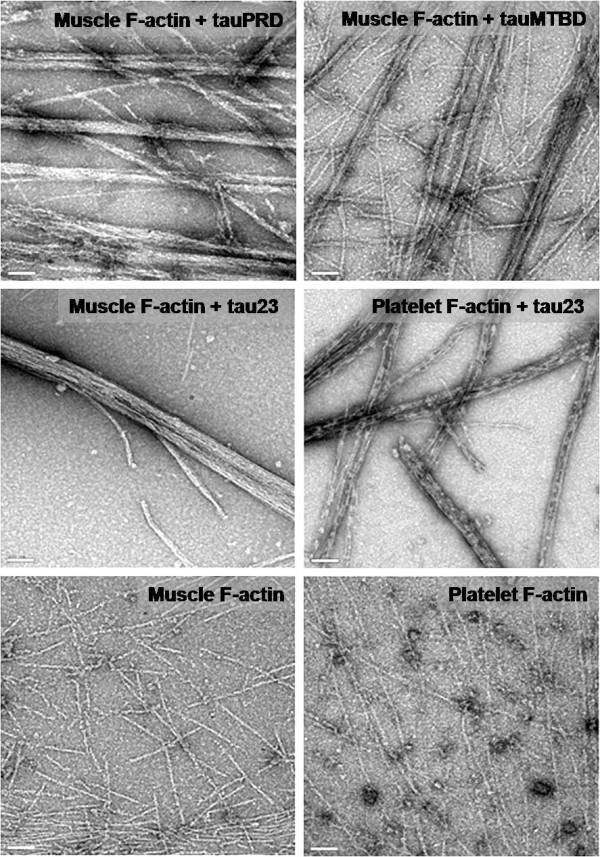
**Electron microscope images of F-actin incubated with tauPRD**. F-actin was incubated with tauPRD, tauMTBD and tau23 as described in Figure 4 and visualized with electron microscope. F-actin alone was shown as negative control. Bars in panels are 100 nm.

Electron microscopy was used to clarify whether tauPRD is able to induce F-actin bundles. Significantly, tauPRD was found to be bound to skeletal muscle F-actin, leading to bundle formation (diameter 38.53 ± 12.32 nm, n = 30) as shown in Figure 9. TauMTBD was used as a positive control. Incubation of tauMTBD with F-actin resulted in bundles (diameter 28.80 ± 8.43 nm, n = 30) but a few filaments remained (diameter 8.61 ± 1.29, n = 30). Tau induced bundling of muscle and platelet F-actin (diameter 54.84 ± 31.84 nm, n = 30). A few actin filaments were observed in the presence of tauPRD or tau. In the absence of tau and the mutants, however, F-actin from muscle tissue or platelets did not form bundles and remained as filaments (diameter 8.58 ± 2.21 nm, n = 30).

### TauPRD induces F-actin bundles at physiological ionic strength

**Figure 10:**
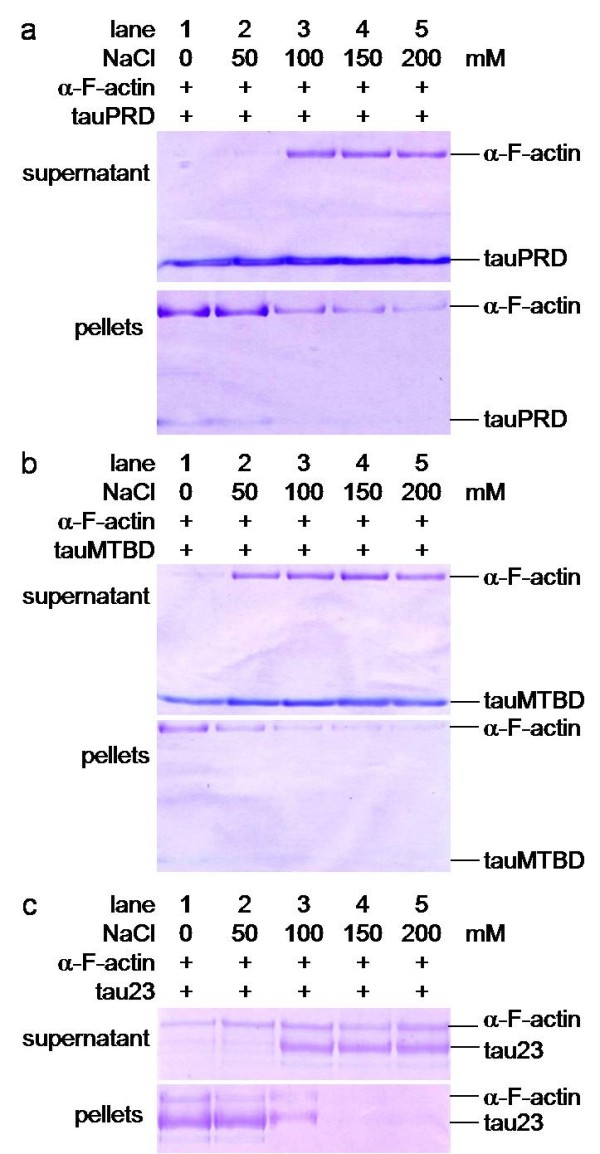
**Low speed Co-sedimentation assays of tauPRD in the presence of F-actin in the presence of NaCl**. Conditions were the same as those in Figure 4, except that F-actin was used instead of G-actin. F-actin was incubated with tauPRD, tau or tauMTBD at different concentrations of NaCl, and precipitates were electrophoresed on SDS-PAGE gels as indicated.

To clarify whether tau interacts with actin in the presence of salts under physiological conditions, we added different concentrations of NaCl to the reaction buffer. After incubating F-actin with tau, low speed co-sedimentation assays showed that the amount of F-actin and tau in the pellets decreased as the concentration of NaCl increased (Figure 10). Similar results were obtained when tauMTBD or tauPRD were employed instead of tau. Under the experimental conditions used, F-actin alone, or with BSA, showed no significant protein precipitates (Additional file 2-b, c). Likewise, precipitates were not detected when tauPRD, tauMTBD, tau or BSA were used in the absence of actin as controls.

**Figure 11:**
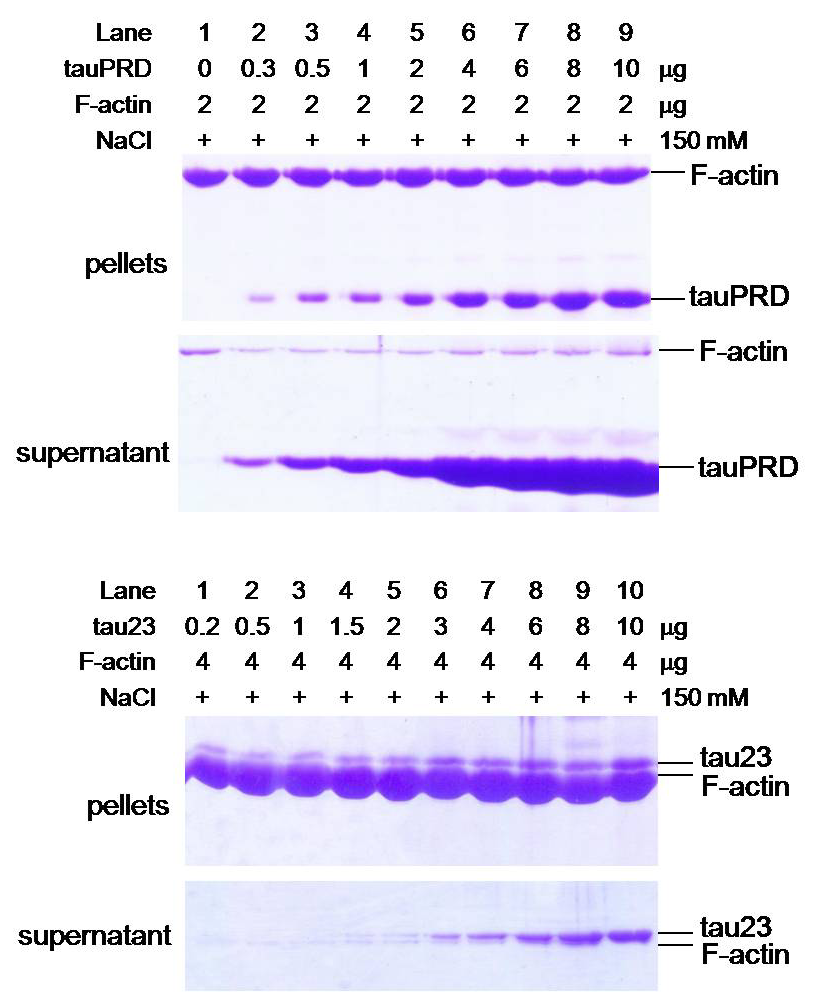
**High speed co-sedimentation assays of tauPRD and F-actin in the presence of NaCl**. F-actin (at a constant amount) was incubated with different amounts of tauPRD (panel a) and tau (panel b) in binding buffer with 150 mM NaCl at 37°C for 40 min to mimic physiological ionic strength and then centrifuged (100,000 g, 4°C, 60 min). The pellet and supernatant were electrophoresed on 12% SDS-PAGE gels.

It is well known that low-speed centrifugation (25,000 g) can precipitate actin bundles but not actin filaments. A few bundles associated with tau could be observed at physiological ionic strength (150 mM NaCl) as shown in Figure 10. We used high-speed centrifugation to further investigate the interaction between tauPRD and F-actin at physiological strength. As shown in Figure 11 (panel a), tauPRD still co-sedimented with actin in the presence of 150 mM NaCl. Under the same conditions, association of tau with F-actin was also visualized (panel b, Figure 11).

**Figure 12:**
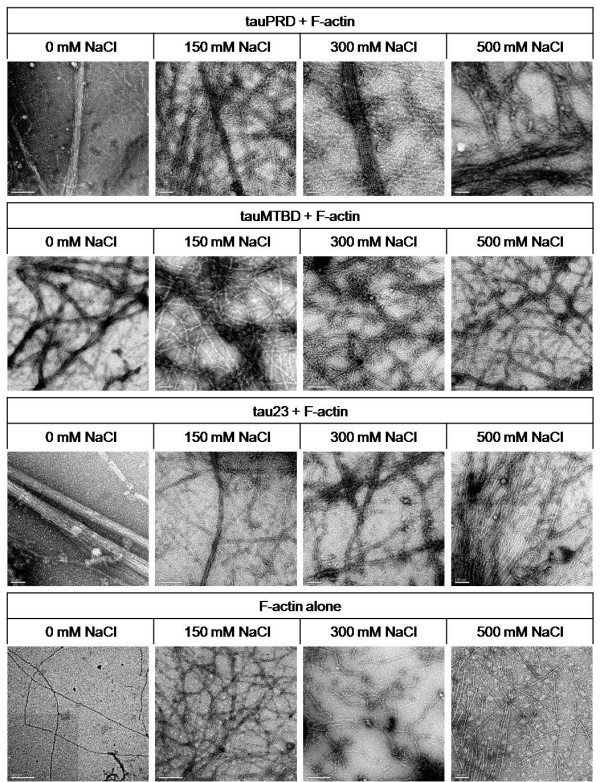
**Electron microscopy images of F-actin incubated with tauPRD in the presence of NaCl at different concentrations**. Conditions were the same as those in Figure 7, except that different concentrations of NaCl were added to the reaction mixtures of F-actin and tauPRD (tau or tauMTBD) as indicated. The bar in each panel represents 200 nm.

Electron microscopy was employed to examine the morphological details of interactions between F-actin and tau or its mutants in the presence of different concentrations of NaCl. As shown in Figure 12, bundles of F-actin could still be observed in 150 mM or higher concentrations of NaCl. The diameter of the actin bundles (39.00 ± 5.86 nm) was similar to those present in the absence of NaCl (38.60 ± 7.28 nm). When NaCl concentration was increased to 300 mM, bundles and some filaments were observed to co-exist in the same visual field. As shown in Additional file 3, the diameter of the bundles was 30.97 ± 8.43 nm and that of the filaments was 8.33 ± 1.29 nm, similar to those present in the absence of NaCl (8.40 ± 1.65 nm). Even when the salt concentration was as high as 500 mM, bundles of F-actin (30.46 ± 6.30 nm diameter) could still be seen in the presence of tauPRD. That is to say that tauPRD was able to promote the assembly of F-actin into bundles at high concentrations of NaCl, but that the diameter of these bundles was somewhat lower than that of those formed in the absence of NaCl. Under our experimental conditions, similar results were obtained for tau and tauMTBD, as indicated in Figure 12.

F-actin alone as a control did not assemble in the presence or absence of NaCl but existed in filaments of similar diameter (8.40 ± 1.65 nm ~9.95 ± 2.09 nm) (Additional file 3). On the other hand, some actin globules were observed when the NaCl concentration was increased to 500 mM, suggesting that actin filaments started to collapse at these high salt concentrations. Tau, tauPRD or tauMTBD appeared as particles without any filaments or bundles in the absence of actin under these experimental conditions (Additional file 4).

## Discussion

Studies on the interaction of tau with actin have generally focussed on the relationship between MTBD and alpha-actin [7,9]. Here we show that the proline-rich domain of tau is also capable of binding to actin, promoting G-actin assembly and F-actin bundling. Our results indicate that: (1) tau is still able to interact with actin after the microtubule binding domain has been deleted (mutant tauΔMTBD); (2) the isolated proline-rich domain alone was able to associate with actin; (3) the tauN, tauC truncation mutants, and the ΔPBD&MTBD deletion mutant showed no interaction with actin; and (4) the isolated proline-rich domain was still able to induce F-actin bundling.

Compared to the microtubule binding domain, the proline-rich domain of tau is a relatively less-well characterized domain. However, evidence has shown that this domain is functionally important and participates in multiple biological processes. PRD has been demonstrated to be an indispensable domain for tau's stabilization of microtubules [20,21]. Further work has shown that PRD may interact with the N-terminal region from another tau molecule to form dimers [22]. Tau was reported to associate with the SH3 domain of fyn and src via its proline-rich domain [23]. Here we show another function of proline-rich domain. As shown in the results above, PRD binds to actin with a lower affinity than native tau (Table 2). The value of Kapp for the complete protein is much lower than that for either tauPRD or tauMTBD. This suggests again that both PRD and MTBD are involved in interactions with actin.

To investigate whether tau or its mutants binds with actin under physiological ionic strength, as suggested by Roger and colleagues [24], we observed the interaction of tau with actin at different concentrations of NaCl. Our results showed that the interaction between tau and actin decreased significantly with increasing ionic strength. However, high ionic strength could not eliminate the interaction completely. At physiological ionic strength, a relatively weak interaction was still observed using high speed co-sedimentation and electron microscopy. We consider high-speed co-sedimentation and electron microscopy are more sensitive and accurate methods than low-speed co-sedimentation. Our results indicate that interactions between F-actin and tau, tauPRD and tauMTBD persist at physiological ionic strength.

Tau and MAP2c are two major microtubule binding proteins that are considered to be potential cross-linkers between microtubules and actin microfilaments. However, the interaction of MAP2c with actin may be different in nature. Konati and colleagues found that tau and MAP2c have different behaviour when studying their effect on actin filament viscosity [11]. Compared with MAP2c, the binding of tau to F-actin was relatively weak at physiological ionic strength [22]. According to Yamauchi and co-workers [8], phosphatidyl-inositol completely disrupts MAP2c-induced bundles. However, tau-induced actin bundles are unaffected by phosphatidyl-inositol. These reports indicate that tau and MAP2c behave differently in their interactions with cytoskeletal components.

Tang and coworkers have proposed the polyelectrolyte theory for F-actin bundling similar to DNA condensation [25]. The general behaviour is dictated by the polyelectrolyte nature of F-actin, which causes a class of nonspecific binding by ligands that carry several net positive charges including divalent metal ions and basic polypeptides. Such bundling is induced by electrostatic force, and usually does not require a specific binding site. Tau is a positively charged protein, with a PI of 9.39 (predicted with Lasergene). Moraga and co-workers proposed that electrostatic forces are involved in the interaction between actin and a tau fragment containing a repetitive sequence from the MTBD domain, because selective carbamoylation resulted in a complete loss of the peptide induction of actin bundles [9]. We have shown that the interaction of tau with F-actin is influenced strongly by ionic strength.

These reports provide evidence that tau may act like polycations to induce F-actin to form bundles. The charge distribution in tau is depicted in Additional file 5 which shows that MTBD and PRD are both highly positively charged, while the N-terminal and C-terminal regions are neutral or negatively charged according to Wang and coworkers [26]. We hypothesize that electrostatic force is the basis of the interaction between tauPRD and actin. The relatively weak and nonspecific nature of the electrostatic forces between tau and F-actin does not necessarily mean that the interaction is of no relevance *in vivo*. Filament concentration could be higher than we can achieve *in vitro*. Besides, several reports have confirmed the physiological importance of this interaction [4,13,14].

## Conclusion

In this work, using co-sedimentation assays and solid phase assays we have shown that the proline-rich domain (PRD) of tau binds with G-actin and F-actin. The PRD domain induced G-actin to form filamentous actin and promoted F-actin to form bundles as observed under both atomic force microscopy and electron microscopy. The promotion of actin bundles in the presence of PRD was also observed in the presence of NaCl under physiological conditions. According to the results presented here and other reports in the literature on MTBD associations with actin, it is suggested that both PRD and MTBD are involved in the association of tau with actin.

## Methods

### Construction of mutants

Constructs of tau mutants (tauN, tauPRD, tauMTBD, tauC, tauΔMTBD and tauΔPRD&MTBD) were prepared by PCR or megaprimer PCR amplification [27] using human tau23 clones as templates. Primers used are listed in Additional file 1. They were then subcloned into the prokaryotic expression vector pET-28a(+) (Novagen, Germany) as NcoI and XhoI fragments. These five mutants each contained a His-tag at the C-terminus. The clones were transformed into *E. coli *BL21 (DE3) cells after their nucleotide sequences had been confirmed by sequencing.

### Expression and purification of tau and its mutants

The tau mutants with His-tags were purified with Ni-NTA resin columns (QIAGEN, Holland) according to the manufacturer's instructions except that cell lysates were boiled for 5 min and centrifuged before loading on the resin. Protein samples were concentrated and then purified further with HiTrap desalting columns (Amersham Pharmacia Biotech, Switzerland). Each purified mutant exhibited a single protein band on Tris-Tricine gels (Additional file 6-a, b). Low molecular weight protein markers (SIBAS, Shanghai, China), mixed with aprotinin (MW 6,500), were used as molecular markers.

The shortest isoform of human tau (tau23) was purified with Q-Sepharose and SP-Sepharose chromatography (Amersham Pharmacia Biotech, Switzerland) as described by Goedert and Jakes [28]. Protein concentrations were measured with BCA protein assay kits (Pierce, USA).

### Western blotting of tau or its mutants

Tau mutants were run on Tris-Tricine gels. The bands on the gels were verified by staining with monoclonal anti-His antibodies (Novagen, Germany) (Additional file 1-b). Tau was run on a 12% SDS-PAGE (Additional file 6-a) and verified by staining with tau-13 antibodies (Santa Cruz, USA). All proteins and mutant peptides employed in this work showed single bands on SDS-PAGE gels or Western blots (Additional file 1).

### Actin preparation

Rabbit skeletal muscle global alpha actin was purified as described by Spudich and Watt [29]. To improve purity, actin was assembled in a buffer containing a low ATP concentration (0.2 mM) during purification. Human platelet G-actin (a mixture of 85% beta and 15% gamma isoforms) was obtained from Cytoskeleton Inc. and was resuspended in buffer A (2 mM Tris-HCl pH 8.0, 0.2 mM ATP, 0.5 mM beta-mercaptoethanol, 0.2 mM CaCl_2_, and 0.005% NaN_3_). G-actin proteins were stored at -70°C after freezing in small volumes in liquid nitrogen. These samples were thawed rapidly with gentle agitation under running water at room temperature [30]. Both skeletal muscle and platelet G-actin showed single bands on SDS-PAGE gels (Figure 2 and Additional file 6). To obtain F-actin, G-actin was polymerized in a buffer containing 100 mM KCl, 2 mM MgCl_2 _and 0.2 mM ATP at 25°C for about 90 min followed by centrifugation (80,000 g, 4°C, and 3 h). The pellet was resuspended in buffer F (100 mM KCl, 1 mM MgCl_2_, 0.1 mM CaCl_2_, 0.2 mM ATP, 1 mM Tris-HCl, pH 8.0, 1 mM NaN_3_).

### Solid phase assays

Experimental conditions were as described by Farias and co-workers [5] with some modifications. The protein (skeletal muscle G-actin, platelet G-actin, skeletal muscle F-actin or platelet F-actin, 5 μg/ml, 50 μl, in 20 mM Tris pH 8.0 buffer) was coated on 96-well microtiter plates (Costar, USA), and incubated at 37°C for 2 h to allow adhesion to the polystyrene surface. After washing three times with PBST (PBS containing 0.2% Tween 20), the sites were saturated by incubation with 200 μl of blocking agent (PBS containing 5% non-fat milk) at 37°C for 30 min. Wells were then washed with binding buffer (50 mM Hepes containing 0.5 mM EGTA and 0.5 mM MgCl_2_, pH 7.5) and 50 μl of different concentrations of tau or its mutants were added. After incubation at 37°C for 45 min, wells were washed with PBST. Primary antibodies (tau-13, Santa Cruz, USA) for tau, and an anti-His-Tag monoclonal antibody (Novagen) for tau mutants were added and incubated at 37°C for 40 min. Plates were washed with PBST before the addition of the second antibody (1:1,000 dilution, 100 μl) labelled with horseradish peroxidase (HRP), and incubated at 37°C for 30 min. After the wells were washed with PBST, binding of actin with tau was detected using 100 μl of TMB buffer (6 μg/ml TMB, 0.045% H_2_O_2_, 0.1 M PB, pH 6.0) for 10 min, and the reaction was terminated with 50 μl of 2 M H_2_SO_4_. The binding of tau with actin was recorded by measuring the net change in absorbance at 450 nm by using an automatic solid phase assay plate reader (Thermo, USA). Correas and co-workers[7] did not add excess ATP to the reaction in their study of the interaction of G-actin with tau. Similarly, Roger and coworkers [24] did not use excess ATP either in studying the bundling of tau with F-actin. Furthermore, Sattilaro and colleagues [31] have reported that formation of MAP-2-actin bundles is inhibited by millimolar concentrations of ATP. Thus, in this work, excess ATP was not used in the reaction of tau with G-actin or F-actin.

### Co-sedimentation assays

Tau or its mutants was incubated with G-actin or F-actin (from skeletal muscle or platelet) in binding buffer in the presence or absence of NaCl (50 - 500 mM) at 37°C for 40 min, and then centrifuged at either 25,000 g for 30 min or 100,000 g for 1 h at 4°C. The pellet was resuspended and then boiled for 5 min, followed by electrophoresis on SDS-PAGE gels.

### Atomic force microscopy (AFM) analysis

G-actin, tau and its mutants were incubated in binding buffer at 37°C for 40 min, and G-actin alone and tau alone were used as controls. Samples were diluted 2 - 20 times with 20 mM Tris-HCl (pH 8.0). A 10 μl drop of the protein sample was deposited on freshly cleaved mica, allowed to stand for 5 min in air, and then washed with three 200 μl aliquots of buffer solution before drying for 4 min in a stream of nitrogen. Tapping mode AFM was performed using a Nanoscope IIIa Multimode-AFM (Veeco Instruments, USA) under ambient conditions. Silicon tips (TESP, Switzerland) with a resonance frequency of about 250 kHz were used at a scan rate of 1-2 Hz. Once the tip was engaged, the set point value was adjusted to minimize the force exerted on the sample while maintaining the sharpness of the image.

### Electron microscopy

Tau (or its mutants) and F-actin were incubated in binding buffer in the presence or absence of NaCl (50 - 500 mM) at 37°C for 40 min. To observe the interaction of F-actin with tau or its mutants, F-actin alone was used as a control under the same conditions. Samples were placed on 300-mesh carbon-coated copper grids for 1 min, washed with H_2_O and negatively stained with 1% uranyl acetate for 1 min. The specimens were examined with a Tecnai 20 electron microscope (Philips, Holland).

## Authors' contributions

HJH and XSW designed and conducted the experiments and analyzed the data. RP and DLW conducted some experiments. MNL prepared tau mutants. RQH designed and supervised the work, and prepared the manuscript for this paper.

## Supplementary Material

Additional file 1 **5'-, 3'- and M-primers used in PCR or megaprimer PCR amplification**. Click here for file

Additional file 2 **Controls for co-sedimentation assays of actin incubated with tauPRD or tau**. Conditions were the same as those for Figure 8, except that tauPRD, tauMTBD and tau were used in the absence of actin for co-sedimentation assays (panel a). F-actin with BSA (panel b) and actin alone (panel c) were employed as controls. Click here for file

Additional file 3 **Diameters of bundles and filaments of F-actin in the presence of tauPRD, tauMTBD and tau, determined using electron microscopy**. Click here for file

Additional file 4 **Electron microscopic images of tauPRD, tauMTBD and tau**. TauPRD, tauMTBD and tau were used as controls in the absence of actin. Click here for file

Additional file 5 **Analysis of the charge distribution of tau40**. Arg and Lys are regarded as positively charged, and Asp and Glu as negatively charged. Thus, for the purposes of calculation, these four residues were considered as the charged components. Sequentially from the N- to the C-terminus, a window of 19 amino acid residues as a group was taken to calculate the average charge as described by Wang and coworkers [26]. Different regions of tau40 protein are indicated by different colours. Click here for file

Additional file 6 **Purification of tau and its mutants**. Six truncated tau isolation and deletion mutants were constructed. Primers used were as shown in Additional file 1. Mutants were constructed as indicated (Figure 4). Mutants were expressed in *E. coli *and then purified through a Ni-NTA column. Samples were electrophoresed on a Tris-Tricine gel (panel b). Western blotting of tau mutants using monoclonal anti-His antibodies (panel b). Tau purified by Q-Sepharose and SP-Sepharose chromatography was analyzed by SDS-PAGE and western blotting using tau-13 anti-tau monoclonal antibodies (panel a). Click here for file
