# Supplementary figures and images for: The proline-rich domain of tau plays a role in interactions with actin
Source: BMC Cell Biol. 2009 Nov 8;10:81. doi: 10.1186/1471-2121-10-81 (PMC2784441; doi:10.1186/1471-2121-10-81)

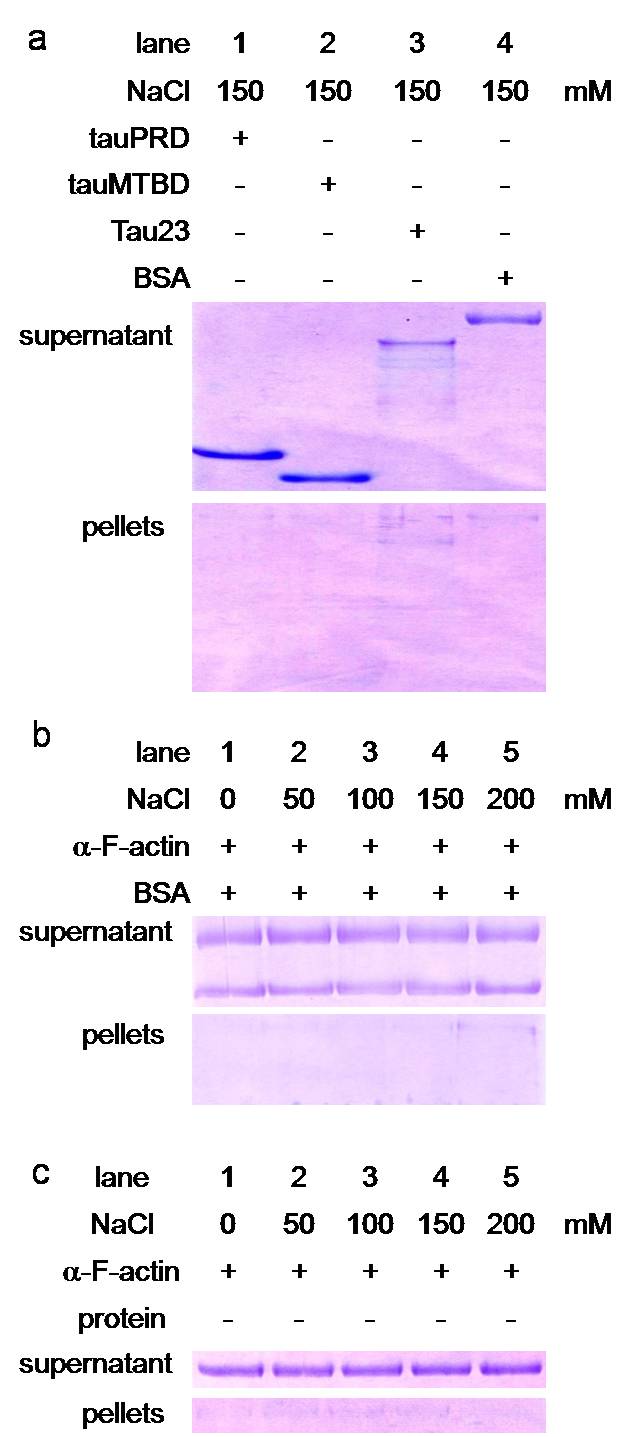

Supplement: Additional file 2 — Controls for co-sedimentation assays of actin incubated with tauPRD or tau. Conditions were the same as those for Figure 8, except that tauPRD, tauMTBD and tau were used in the absence of actin for co-sedimentation assays (panel a). F-actin with BSA (panel b) and actin alone (panel c) were employed as controls. [file 1471-2121-10-81-S2.jpeg]

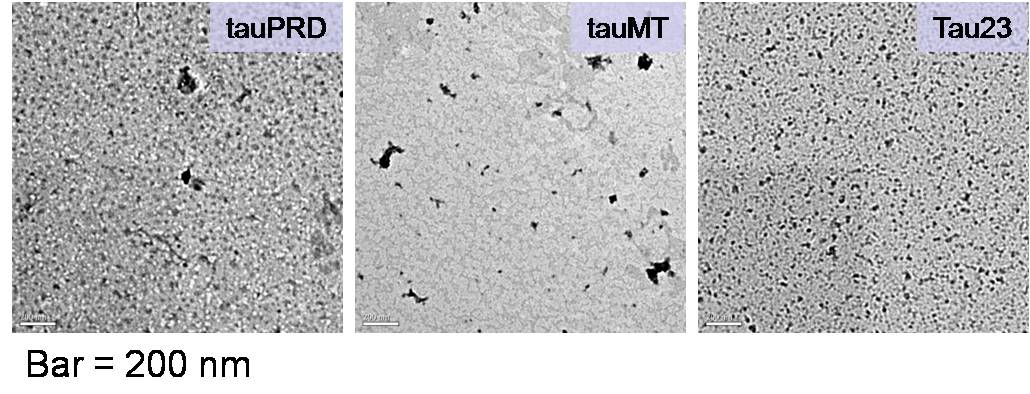

Supplement: Additional file 4 — Electron microscopic images of tauPRD, tauMTBD and tau. TauPRD, tauMTBD and tau were used as controls in the absence of actin. [file 1471-2121-10-81-S4.jpeg]

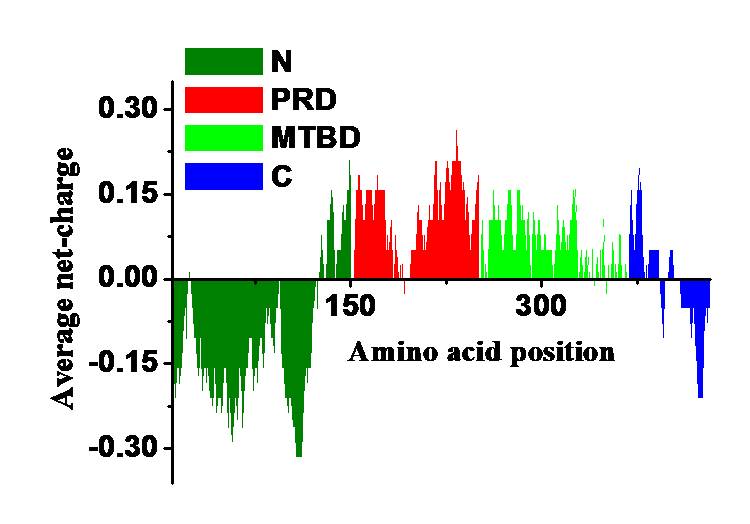

Supplement: Additional file 5 — Analysis of the charge distribution of tau40. Arg and Lys are regarded as positively charged, and Asp and Glu as negatively charged. Thus, for the purposes of calculation, these four residues were considered as the charged components. Sequentially from the N- to the C-terminus, a window of 19 amino acid residues as a group was taken to calculate the average charge as described by Wang and coworkers [26]. Different regions of tau40 protein are indicated by different colours. [file 1471-2121-10-81-S5.jpeg]

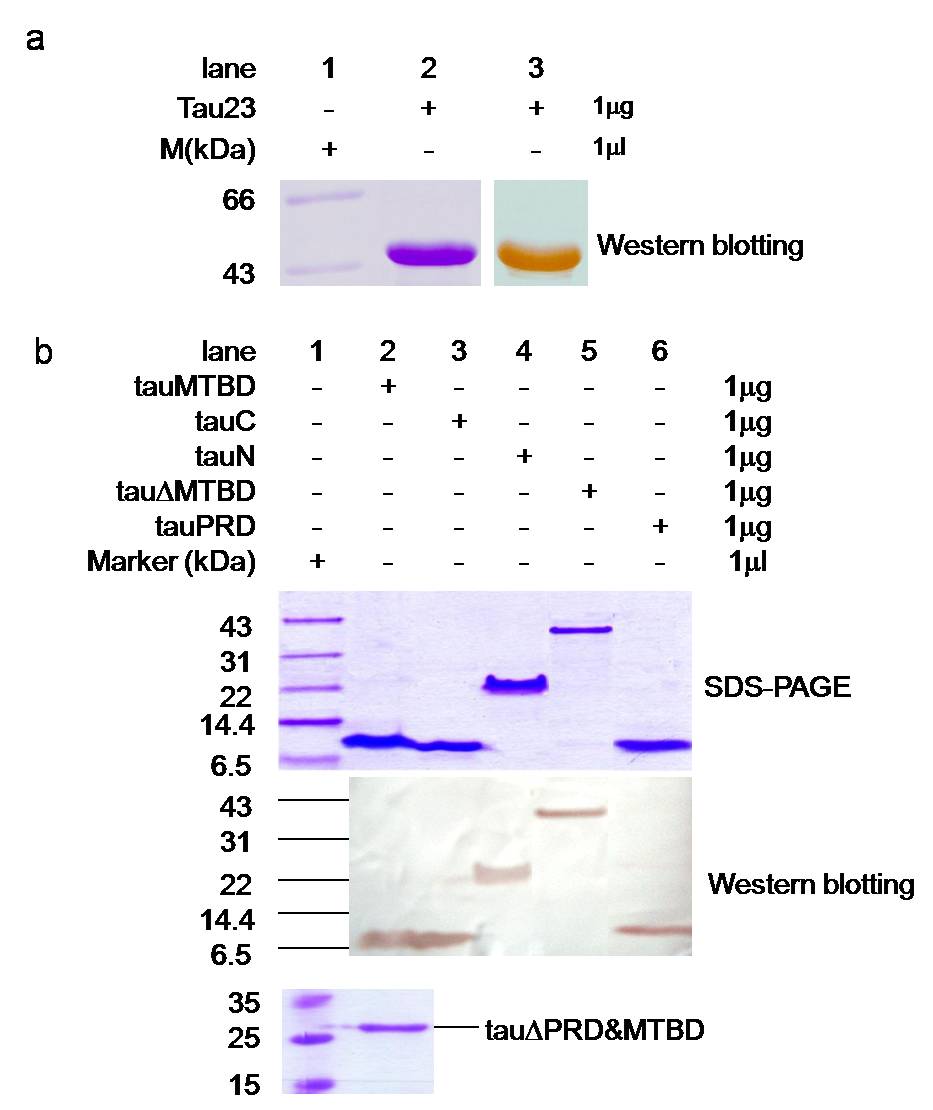

Supplement: Additional file 6 — Purification of tau and its mutants. Six truncated tau isolation and deletion mutants were constructed. Primers used were as shown in Additional file 1. Mutants were constructed as indicated (Figure 4). Mutants were expressed in E. coli and then purified through a Ni-NTA column. Samples were electrophoresed on a Tris-Tricine gel (panel b). Western blotting of tau mutants using monoclonal anti-His antibodies (panel b). Tau purified by Q-Sepharose and SP-Sepharose chromatography was analyzed by SDS-PAGE and western blotting using tau-13 anti-tau monoclonal antibodies (panel a). [file 1471-2121-10-81-S6.jpeg]
